# Deficient Knowledge on Hepatitis B Infection in Pregnant Women and Prevalence of Hepatitis B Surface Antigen Carriage in an Endemic Area: A Review

**DOI:** 10.1155/2012/317451

**Published:** 2012-09-28

**Authors:** Oi Ka Chan, Terence T. Lao, Stephen S. H. Suen, Tak Yeung Leung

**Affiliations:** Department of Obstetrics and Gynaecology, Prince of Wales Hospital, The Chinese University of Hong Kong, Shatin, New Territories, Hong Kong

## Abstract

Hepatitis B infection is a major global health problem. Vertical transmission is the commonest route of spreading hepatitis B virus (HBV) in many endemic areas. In order to control such transmission in Hong Kong, neonatal immunization programme was implemented for more than two decades. A declining prevalence of HBV infection was expected. However, the prevalence remained unabated at around 10% in recent studies. We suspect that one of the explanations of this persistent high prevalence is deficient knowledge on infection with the HBV and its prevention. Our paper gives an overview of the knowledge on HBV infection among Chinese population in both high and low endemic areas and discusses the potential factors that influenced the knowledge on as well as the implication of the sources of information for HBV infection, which was not addressed in previous studies.

## 1. Background

Hepatitis B infection is a worldwide problem with over 350 million carriers [1]. Subjects with chronic hepatitis B virus (HBV) infection are at increased risk of hepatocellular carcinoma, cirrhosis, and chronic hepatitis [2–4]. Chronic infection with HBV is endemic in the Asia-Pacific region and China. Hong Kong, as part of China, is a high-prevalence area for HBV infection according to the WHO definition, and antenatal screening for maternal infection, in the form of screening for hepatitis B surface antigen (HBsAg), is a standard procedure. Hong Kong is one of the first cities that introduced immunoprophylaxis to the neonates in 1983, with combined immunoglobulin and vaccine given to offspring of mothers with positive screening, and this was followed by universal vaccination to all newborn infants from 1988 [5, 6]. Compliance is ensured by means of a vaccination record issued to all children, which has to be checked by teachers at primary and secondary schools (under the enforced free education system) to ensure that incompletion of any vaccination could be remedied. Furthermore, all nonimmune adult residents of Hong Kong have opportunities in obtaining vaccination from various institutions such as universities and nongovernment organizations. Good compliance with the vaccination, especially in a nationwide government-initiated public health programme as implemented in Taiwan, has resulted in a decline in the HBV carrier rate in children from 10% to <1% and reduction in mortality from fulminant hepatitis and hepatoma in children [7, 8]. Yet in the past four decades, studies on maternal HBV infection in Hong Kong yielded the prevalence of 6.6% in 1976 [9], 7.4% in 1983 [10], and 10.0% in 1996 [5], and which has remained unabated at around 10% in the most recent studies [11, 12]. Therefore, the persistence of a high and apparently rising prevalence of HBV infection was unexpected. We suspect that one of the explanations of this persistently high prevalence is deficient knowledge on infection with the HBV, especially regarding its prevention in horizontal transmission, in the fertile female population.

## 2. Studies on Knowledge

 There are few reported studies on the knowledge of HBV infection among pregnant women, which can be taken as proxy for the fertile females among the general population. To address this issue, we have conducted a survey to examine the knowledge of HBV infection in a nonselected cohort of Chinese pregnant women attending our antenatal clinic in 2008 [13]. We found insufficient knowledge on HBV infection in various aspects which were similar to the findings in all other studies in the Chinese immigrant populations in some low endemic areas [14–20]. It is noteworthy that between 27% and 75% of the studied subjects realized that HBV infection can be a lifelong condition, and 75% and 60% of them knew that HBV infection is associated with cirrhosis and liver cancer, respectively. The findings of the aforementioned studies are summarized in Table 1.

## 3. Knowledge on Vertical Transmission and Its Prevention

### 3.1. Hepatitis B Neonatal Vaccination Programme

 Vertical transmission of HBV from an infected mother to her infant is a major source of infection in many endemic areas [5, 10, 23–27]. In order to control the vertical transmission of HBV, neonatal immunisation programmes involving the use of immunoglobulin and hepatitis B vaccine have been adopted in many countries [28, 29]. In Hong Kong, this programme was introduced in 1983, following a prospective randomized study that had proved its efficacy [10, 30]. From 1983 to 1988, this programme was selective in that only neonates born to mothers with chronic HBV infection, as reflected in their positive HBsAg status, received immunoglobulin and a triple dose vaccination at the time of birth [5]. From November 1988, this programme became universal and covered all neonates born to both HBsAg positive and negative mothers as a government-initiated standard public health preventive measure, and a free vaccinations package, including that for HBV, was provided to all newborn infants in the Government Maternal and Child Health Centres under the Department of Health [6, 31]. Nowadays, the universal immunization programme is available to the infants of all local residents. The effectiveness of universal immunization has been proven by the demonstration of reduced prevalence of childhood HBV infection and hepatocellular carcinoma in a number of endemic areas [2, 8, 10, 32, 33].

 Nevertheless, despite the fact that the HBV vaccine and the immunization prgramme have been introduced since the 1980s, the knowledge on perinatal transmission of HBV was quite variable among different Chinese populations, ranging from 40% to 91%. Knowledge was most deficient amongst the Chinese immigrants in New York city, where only 40% of the surveyed subjects could give a correct response [19]. The results of these studies suggested that further efforts should be made in educating all Chinese women in the reproductive age group irrespective of their place of residence about vertical transmission of HBV infection and its prevention by the neonatal immunization programme.

## 4. Knowledge on Horizontal Transmission and Its Prevention

### 4.1. Horizontal Transmission

 In addition to perinatal transmission, HBV can spread through sexual intercourse and contact with infected blood products through transfusion, sharing of needles and unsafe injecting equipments. Transmission through sexual contact is documented as a major route of spread of HBV in countries with low and intermediate endemicity [24, 34], while blood transfusion and unsafe injection are main sources of HBV transmission in many developing areas [35–37]. It has been shown that many Chinese people were not aware of the role of horizontal transmission of HBV, especially regarding the transmission of HBV through sexual contact. Only 40–65% of surveyed subjects knew that HBV could be sexually transmitted [13–22]. This deficiency in knowledge is most likely related to the oversight of not including HBV infection as one form of sexually transmitted diseases in public health promotion and educational materials, a situation that should be rectified in all places irrespective of the local prevalence of HBV infection.

However, another important aspect of deficient knowledge in Asian and Chinese communities worldwide is the risk of horizontal transmission through means others than sexual intercourse, because of the unique culture associated with the Chinese and their family settings, especially the sharing of food and eating utensils. Earlier studies have shown the presence of HBsAg in several body fluids such as saliva, semen, and urine [38–41]. Since then, HBV transmission from saliva had also been described in some studies [42–45]. Furthermore, a local case of HBV transmission by human bite had been reported by a research group in recent year [46] and suggested that human bite is another route of HBV transmission. Indeed, HBV infection from saliva contact through bites or other wounds on the skin and open mouth ulcer, and as through prechewing of food from infected persons, has been reported repeatedly [46–51]. As premastication of food by the mother or grandparents before the food is fed to the infant and the shared use of chopsticks, communal eating, and sharing of utensils are still common practices in Chinese families, transmission by infected saliva could be a serious yet overlooked means of transmission, especially when the exposure by the susceptible subject would be continuous and prolonged. Indeed, even in some recent studies [17, 18, 20], 11–43% of the Chinese subjects surveyed thought that HBV could not be transmitted by the sharing of eating utensils. Furthermore, HBV can survive for weeks outside the body, and it can be found on contaminated inanimate objects, such as toothbrushes and razor [52–55]. In previous studies, 41% to 86% of the subjects knew that sharing razor or toothbrush, tattooing or body piercing (37%), eating food that has been prechewed by an infected person (69–82%), sharing of needles (52–83%), or exposure to blood or blood products (65–90%) could allow HBV to spread from an infected person to susceptible individuals. In our study [13], 50.2% of the subjects thought that HBV could be transmitted through exposure to body fluids such as saliva and urine. While horizontal transmission through the contact with infected urine was an unlikely or remote cause of infection among the fertile women in our society, contact with saliva, such as from kissing and even from orally sexual activity, is probably much more common. Further studies are warranted to clarify the role of horizontal transmission by infected saliva, but in the meantime, it would be prudent to remind the community not to share eating utensils and to avoid all the aforementioned practices in the family setting for hygiene considerations.

On the other hand, in line with other researchers, we have also found erroneous knowledge amongst our subjects. In our study, only 24% of the subjects recognized that HBV is not transmitted by the oral-fecal route, which was consistent with the findings of other studies in the same area [21, 22]. This suggested that there was confusion between hepatitis A and hepatitis B amongst the general public.

### 4.2. Prevention

Only a few studies had examined the knowledge on prevention of HBV transmission, and the majority of the respondents knew that HBV transmission could be prevented by hepatitis B screening and vaccination (62–95%). In Hong Kong, vaccination programmes are provided to all nonimmune adults who requested vaccination by institutions such as universities, and nongovernment organizations such as the Family Planning Association, and by general practitioners [5, 56]. With increased public awareness of HBV infection over the past two decades, the rate of HBV vaccination uptake at their own expense amongst the pregnant women in Hong Kong increased from 13% in 1996 [5] to 33% in 2008 [56]. Nevertheless, there is much room for improvement and catch-up vaccination or booster doses should be offered to all individuals without confirmed immunity to HBV whenever such individuals are identified.

Among the public, less is known about the risk and importance of horizontal transmission; although health education pamphlets, posters, and television advertisements have explained about the risk of HBV in needle-sharing, acupuncture and tattooing, it is not certain to what extent do the general and obstetric population realizes the importance of such information. In Hong Kong, safe injection practice is performed in the clinical settings without the reuse of syringes and needles in both public and private hospitals, and our pregnant women are at minimal risk of HBV infection via routine antenatal blood taking or drug injection. However, acupuncture and beauty treatments were risk factors in the spread of HBV infection [57, 58], but the safe use of needles in acupuncture and beauty treatments cannot be guaranteed, even though disposable needles are available. In Mainland China, the injection practice, especially in some rural areas, remains questionable. The difference in rate of HBV infection between our local women and the immigrants from Mainland China could be partly explained on the differences in these practices, but this issue needs to be explored in further studies.

At the same time, there remains a substantial portion of our subjects with erroneous knowledge on the prevention of HBV transmission, as only 14% of pregnant respondents knew that neither a balanced diet and vitamin C consumption, nor regular exercise, nor getting enough rest can prevent HBV infection. However, in another recent study amongst the general public, up to 98% of the respondents knew that balanced diet and vitamin C could not prevent HBV infection [21]. Such discrepancy of knowledge found in the same area could be explained by the fact that subjects from the study of Chung et al. [21] were generally better educated, while the pregnant women attending our antenatal clinic were deficient in the knowledge about prevention of HBV infection.

## 5. Factors Influencing Knowledge among the Fertile Women

### 5.1. Immigration

 In some low endemicity countries such as the United States and Canada, immigration from highly endemic areas such as Southeast Asia has been the major contributor to the horizontal transmission from the carriers to other susceptible individuals, leading to an increasing prevalence of chronic HBV infection [16]. In Hong Kong, a high influx rate of immigrants and visitors from Mainland China in the past decade is thought to be an important contributing factor to the persistence of a high prevalence rate of chronic infection in pregnant women. We had demonstrated an association between status of residency and the HBV carriage rate (5.7% for locally born residents versus 14.2% for immigrants and nonresidents), with the uptake of hepatitis B vaccine (36.0% for local residents versus 22.3% for nonlocal residents), and poor knowledge on HBV transmission and prevention (aOR ranged 1.63–2.01 for new immigrants and 1.65–1.81 for nonresidents as compared with locally born residents) [12, 13, 56].

### 5.2. Sources of Information

In Hong Kong, the Viral Hepatitis Preventive Service has been launched by the Department of Health in Hong Kong in July 1998, conducting epidemiological surveillance and distributing information through various channels to the general public. There are also other local sources or channels available for the dissemination of information regarding HBV infection in Hong Kong, such as healthcare professionals, mass media, the Department of Health website [59], and educational pamphlets. In our community, the mass media are the most preferred channels for disseminating educational materials and information to general public at the community level, because of their popularity [60, 61]. Mass media, such as televisions and radios, are available to almost every household in Hong Kong, and free news channels are available in public transports such as buses and the mass transit system. Furthermore, many newspapers are distributed free of charge and internet services are also easily accessible in the community. We have therefore examined the sources of information on HBV infection and the roles of various channels in the dissemination of information with respect to the level of knowledge [13], which was not addressed in previous studies. Our aim was to determine whether accessibility and utilization of the various sources of information could have contributed to deficient or incorrect knowledge among our respondents.

**Figure 1:**
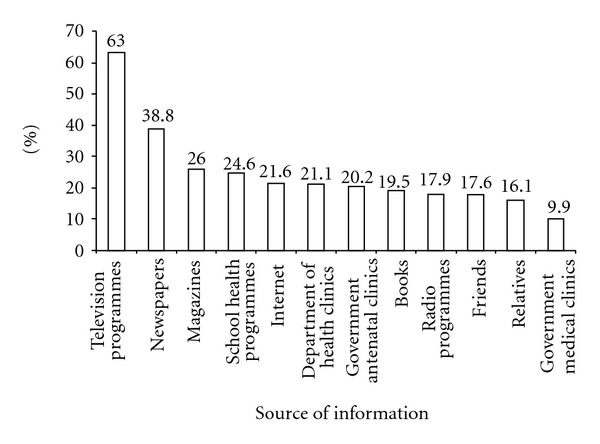
The distribution of the source of information on HBV knowledge.

We found that mass media is the most important source of hepatitis B knowledge among our pregnant women, with the two leading sources of knowledge being television programmes (63.0%) and newspapers (38.8%) (Figure 1). Similar to television programmes, radio programmes are also one of the most rapid means of disseminating information in real time to the public and of increasing their awareness and knowledge of HBV infection, but only a minority (17.9%) indicated that radio programmes were useful in learning about HBV infection. Each of the other sources accounted for 26% or less. Medical and health care services were among the lowest with the poorest source being government medical clinics (9.9%). What was most disappointing was that around 80% of the respondents did not receive any information about HBV infection from the medical and healthcare services or medical professionals, which should have been the optimal channel to disseminate correct medical information. In Hong Kong, primary medical care is provided at a nominal charge to all local residents, and many charitable organizations provide free medical care to those who cannot afford the outpatient charges. Furthermore, government maternal and child health centers and the Family Planning Association also provide service to all without restriction. The mechanisms of this failure remain to be established, but it was possible that when these women sought help from such services, they were focused only on their complaints or problems at the time. As well, the need for specific health education on the common endemic infections in our territory, such as HBV infection, might not have crossed the minds of the health care professionals at the time due to the assumption that such as information should have been readily available.

**Figure 2:**
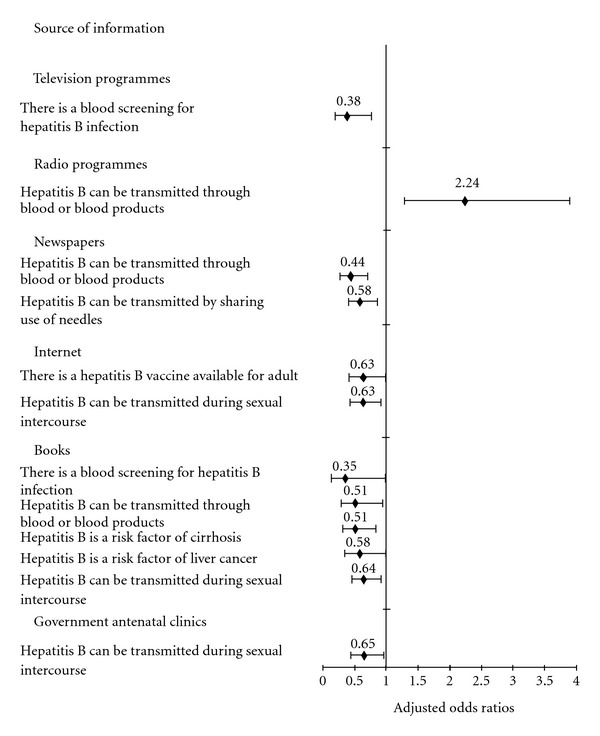
The adjusted odds ratios and 95% CI of the source of information for incorrect responses to items.

As we have noted that incorrect or misleading messages have been provided to our subjects, the effects of the various channels of dissemination of the information on HBV infection were assessed by multivariate logistic analysis and the adjusted odds ratios of the source of information for incorrect response on individual item were presented in Figure 2. After adjusting for the significant sociodemographic, medical, and obstetric factors found in univariate analysis [13], television programmes (aOR 0.38 in 1 item), books (aOR range 0.35–0.64 in 5 items), newspapers (aOR range 0.44 and 0.58 in 2 items), internet (both aORs 0.63 for 2 items), and government antenatal clinics (aOR 0.65 for 1 item) were associated with reduced provision of incorrect knowledge about HBV infection for most items. At the same time, however, radio programmes (aOR 2.24) were also revealed to be the significant source of provision of incorrect information regarding the transmission of HBV through blood or other blood products. We suspect that some of information acquired from this channel might be erroneous or lacking a scientific or medical basis, and this issue should be examined specifically in future studies.

## 6. Conclusion

 Numerous studies on HBV infection, its sequelae, and the various means of prevention have been published in the past three decades. Yet even the latest studies have found areas of deficient or even erroneous knowledge on HBV infection, and we are especially disappointed to learn from the findings in our pregnant women that there is much room for improvement in the provision of appropriate and correct information on HBV transmission and prevention to the public. We suspect that deficient knowledge and misconceptions, especially regarding the various means of horizontal transmission, have probably contributed to the persistently high prevalence of HBV infection in our obstetric population. Social stigma can result from poor knowledge on HBV infection, as is the case in Mainland China. In China, HBV carriers face social discrimination affecting both their life and work as many employers and universities refuse to accept those who were tested positive from the preemployment and preenrolment medical checkup, although according to Ministry of Health of the People's Republic of China, the China Government has decided to legislate against the hepatitis B discrimination recently [62]. On the other hand, appropriate public education could reduce the stigma attached to HBV carriers, as shown in the United States that higher levels of knowledge regarding HBV were associated with lower degrees of stigma [20]. Where resources are limited, targeting women in the reproduction age group for health education would be most cost-effective due to their roles as mothers and care providers to the entire household, so that their possession of correct knowledge on HBV transmission and infection would have the greatest overall impact on the population. More studies on the control of HBV infection through enhanced public health education programmes as an adjunct to any ongoing immunization programme in endemic areas are warranted.

## Figures and Tables

**Table 1:** Summary of hepatitis B knowledge among Chinese population in low and high endemic areas.

First author and year in which study was conducted	Chan et al. [13] 2008	Chung et al. [21] 2009	Leung et al. [22] 2010	Coronado et al. [16] 2005	Cotler et al. [20] 2012*	Taylor et al. [17] 2005	Hislop et al. [18] 2005	Thompson et al. [15] 1999	Thompson et al. [14] 1999	Ma et al. [19] 2004
Study population (*n*)	Pregnant women (*n* = 1623)	General public (*n* = 1982)	General public (*n* = 506)	General public (*n* = 430)	Patients attended routine care (*n* = 201)	General public (*n* = 395)	General public (*n* = 504)	Women (*n* = 147)	Women (*n* = 124)	General public (*n* = 429)
Study location	Hong Kong	Hong Kong	Hong Kong	Seattle, USA	Illinois, USA	Washington, USA	Vancouver, Canada	Vancouver, Canada	Washington, USA	New York, USA
There is a blood screening test for hepatitis B infection (yes)	93	96	—	—	—	—	—	—	—	55
There is a hepatitis B vaccine available for nonimmune adult (yes)	75	79	—	—	—	—	—	—	—	53
HBV can be spread by someone who looks health (yes)	—	—	—	80	50	79	80	68	48	—

Mode of transmission										
Through food or drink (no)	24	53	27	—	31	—	—	—	—	—
Through blood or blood products (yes)	76	82	65	—	90	—	—	—	—	—
During sexual intercourse (yes)	53	59	44	56	60	54	65	56	48	40
Sharing use of needles (yes)	69	83	—	—	—	—	—	—	—	52
During childbirth (yes)	88	58	67	70	91	70	76	—	—	40
Shaking hands with infected person (no)	50	92	—	—	—	—	—	71	58	85
Sharing razor or toothbrush	—	—	41	—	—	55	68	86	68	52
Tattooing or body piercing	—	—	37	—	—	—	—	—	—	—
Sharing eating utensils (yes)	—	—	—	—	57**	84**	89**	—	—	61
Eating food prepared by an infected person (no)	—	—	—	—	—	23	24	41	21	67
Eating food that has been prechewed by an infected person (yes)	—	—	—	—	—	—	—	82	69	—

Prevention of transmission										
Hepatitis B screening and vaccination (yes)	87	92	—	—	95	—	—	—	—	62
Regular exercise and get enough rest (no)	14	41	—	—	—	—	—	—	—	57/74
Balanced diet and vitamin C (no)	14	98	—	—	—	—	—	—	—	76

Sequelae of infection										
Lifelong infection (yes)	—	—	—	38	75	37	45	39	27	—
Cirrhosis (yes)	86	90	—	—	98	75	83	—	—	—
Liver cancer (yes)	83	87	—	71	92	73	81	61	46	72

*Year of paper was published.

**Original answer by authors was “no”.

Each item listed with the correct answer inside parenthesis. Figures were rounded to whole number. Data were presented as percent of correct answer for each item.
